# Antagonism of Transient Receptor Potential Ankyrin Type-1 Channels as a Potential Target for the Treatment of Trigeminal Neuropathic Pain: Study in an Animal Model

**DOI:** 10.3390/ijms19113320

**Published:** 2018-10-25

**Authors:** Chiara Demartini, Rosaria Greco, Anna Maria Zanaboni, Oscar Francesconi, Cristina Nativi, Cristina Tassorelli, Kristof Deseure

**Affiliations:** 1 Laboratory of Neurophysiology of Integrative Autonomic Systems, Headache Science Center, IRCCS Mondino Foundation, via Mondino 2, 27100 Pavia, Italy; rosaria.greco@mondino.it (R.G.); annamaria.zanaboni@mondino.it (A.M.Z.); cristina.nativi@unifi.it (C.T.); 2 Department of Brain and Behavioral Sciences, University of Pavia, via Bassi 21, 27100 Pavia, Italy; 3 Department of Chemistry ‘Ugo Schiff’, University of Florence, Via della Lastruccia 3-13, 50019 Sesto Fiorentino (FI), Italy; oscar.francesconi@unifi.it (O.F.); cristina.nativi@unifi.it (C.N.); 4 Department of Medicine, Laboratory for Pain Research, University of Antwerp, Universiteitsplein 1, 2610 Wilrijk, Belgium; kristof.deseure@uantwerpen.be

**Keywords:** neuropathic pain, trigeminal system, allodynia, TRPA1, TRPV1

## Abstract

Transient receptor potential ankyrin type-1 (TRPA1) channels are known to actively participate in different pain conditions, including trigeminal neuropathic pain, whose clinical treatment is still unsatisfactory. The aim of this study was to evaluate the involvement of TRPA1 channels by means of the antagonist ADM_12 in trigeminal neuropathic pain, in order to identify possible therapeutic targets. A single treatment of ADM_12 in rats 4 weeks after the chronic constriction injury of the infraorbital nerve (IoN-CCI) significantly reduced the mechanical allodynia induced in the IoN-CCI rats. Additionally, ADM_12 was able to abolish the increased levels of TRPA1, calcitonin gene-related peptide (CGRP), substance P (SP), and cytokines gene expression in trigeminal ganglia, cervical spinal cord, and medulla induced in the IoN-CCI rats. By contrast, no significant differences between groups were seen as regards CGRP and SP protein expression in the pars caudalis of the spinal nucleus of the trigeminal nerve. ADM_12 also reduced TRP vanilloid type-1 (TRPV1) gene expression in the same areas after IoN-CCI. Our findings show the involvement of both TRPA1 and TRPV1 channels in trigeminal neuropathic pain, and in particular, in trigeminal mechanical allodynia. Furthermore, they provide grounds for the use of ADM_12 in the treatment of trigeminal neuropathic pain.

## 1. Introduction

Trigeminal neuralgia (TN) is a rare condition characterized by paroxysmal attacks of sharp pain, frequently described as an “electric shock”. Up to 50% of patients with trigeminal neuralgia also have continuous pain in the same territory, which results in greater diagnostic difficulties, higher disability, and lower response to medical and surgical treatments [1]. Three diagnostic categories of TN are identified by the recent classification of headache disorders: Classical (without apparent cause other than neurovascular compression), secondary (caused by an underlying neurological disorder), and idiopathic (no cause is found) [2]. TN has a negative impact on activities of daily living, with up to 45% of patients being absent from usual daily activities for 15 days or more, and one third suffering from mild-to-severe depression [3]. Medications for TN exist, but they are poorly tolerated or ineffective. For this reason, multiple surgical approaches have been developed, but a portion of patients are refractory to both medical and surgical approaches [4,5]. Hence, there is need for further investigation into the mechanisms underlying pain in TN in order to identify new, possibly more effective, therapeutic targets.

In recent years, transient receptor potential (TRP) channels have attracted much attention in the pain field. These channels are non-selective cation channel proteins, widely distributed in many tissues and cell types, localized in the plasma membrane and membranes of intracellular organelles [6]. The TRP ankyrin type-1 (TRPA1) channels, mainly expressed with the vanilloid type-1 (TRPV1), are localized in a subpopulation of C- and Aδ-fibers of neurons located in the dorsal root ganglia (DRG) and trigeminal ganglia (TG) that produce and release neuropeptides, such as substance P (SP), neurokinin A, and calcitonin gene-related peptide (CGRP) [7,8,9]. Many experimental studies, from genetic knockouts to pharmacological manipulation models, reported a critical involvement of TRPA1 channels in different aspects of pain [10] and a role in several models of nerve injury, such as the lumbar spinal nerve ligation [11], and sciatic nerve injury by chronic constriction or transection [12,13,14]. In these models, it was demonstrated that an up-regulation of TRPA1 is associated with mechanical and thermal hyperalgesia, a condition reversed by TRPA1 antagonists [15,16]. In a recent study, Trevisan and colleagues [17] reported that pain-like behaviors are mediated by the TRPA1 channel in an animal model of TN based on the constriction of the infraorbital nerve (IoN) via the increased oxidative stress by-products released from monocytes and macrophages that gather at the site of nerve injury. 

The aim of this study was to further investigate the role of TRP channels in trigeminal neuropathic pain induced by the model of a chronic constriction injury of the IoN (IoN-CCI) [18]. More specifically we evaluated: (i) The modulatory effect of TRPA1 antagonism, by means of ADM_12 treatment, on IoN-CCI-induced allodynia; (ii) the levels of TRPA1 and TRPV1 mRNA in specific cerebral and peripheral areas involved in trigeminal sensitization, with particular attention to changes in expression levels of genes coding for CGRP, SP, and cytokines after TRPA1 antagonism; and (iii) the expression of CGRP and SP proteins in the Spinal Nucleus of trigeminal nerve pars caudalis (Sp5C).

## 2. Results

### 2.1. ADM_12 Effect on Behavioral Response

**Figure 1:**
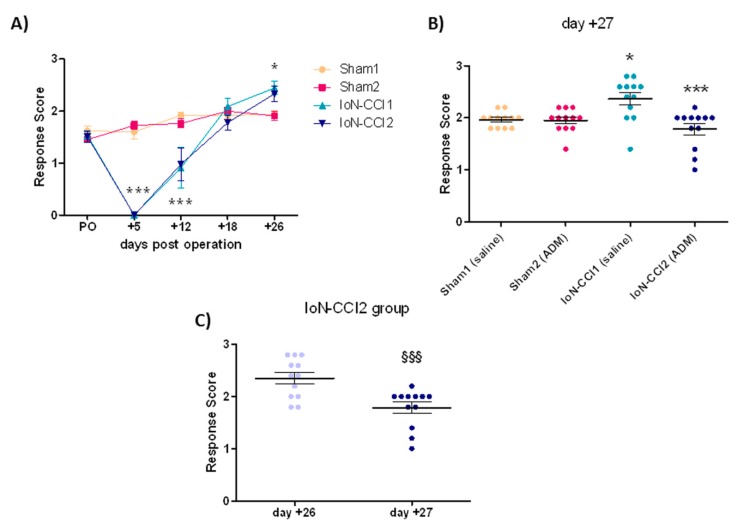
Mechanical stimulation testing (MST): (**A**) Mean response score to Von Frey hair stimulation of the ligated/sham infraorbital nerve (IoN) territory, on pre-operative day (PO) and on +5, +12, +18, and +26 days post operation. Data is expressed as mean ± SEM. Two-way ANOVA followed by Bonferroni post-hoc test, * *p* < 0.05 and *** *p* < 0.001 for chronic constriction injury of the infraorbital nerve (IoN-CCI) groups vs. Sham groups. Drug treatment effect on MST: (**B**) Mean response score to Von Frey hair stimulation on day +27, 1 h after ADM_12 (or saline) treatment. Data is expressed as mean ± SEM. One-way ANOVA followed by Tukey’s Multiple Comparison Test, * *p* < 0.05 vs. Sham1 and Sham2, *** *p* < 0.001 vs. IoN-CCI1. (**C**) Comparison of the IoN-CCI2 group without treatment (day +26) and after ADM_12 treatment (on day +27). Data is expressed as mean ± SEM. Paired Student’s *t* test, ^§§§^ *p* < 0.001 vs. day +26.

In agreement with Deseure and Hans [18], 5 days after surgery, the two groups of rats that underwent IoN-CCI displayed a lack of responsiveness to ipsilateral mechanical stimulation testing (MST) of the IoN territory (Figure 1A). At day 12, the hyporesponsiveness was recovering to be replaced at day +26 by a significant increase in the MST response score as compared to the two Sham groups (Figure 1A). On day +27, the administration of the TRPA1 antagonist treatment in operated rats (IoN-CCI2 group) reduced the response score of the mechanical stimulation compared to the IoN-CCI1 group (injected with saline) (Figure 1B); whereas, ADM_12 treatment in sham-operated rats (Sham2 group) did not change the mechanical response. It is of note that the response to MST in the IoN-CCI2 group was significantly different between day +26 (before ADM_12 injection) and +27 (after drug treatment) (Figure 1C).

### 2.2. ADM_12 Effect on Gene Expression

The expression of *Trpa1*, calcitonin-related polypeptide alpha (*Calca*), and preprotachykinin-A, (*PPT-A*) was evaluated in the TG and cervical spinal cord (CSC) ipsilateral (ipsi) and contralateral (contra) to the IoN ligation, and in the medulla in toto. Because of the strong relationship between TRPA1 and TRPV1 channels [19,20,21], we also investigated the *Trpv1* mRNA expression levels in the same areas.

#### 2.2.1. *Trpa1* mRNA Expression

**Figure 2:**
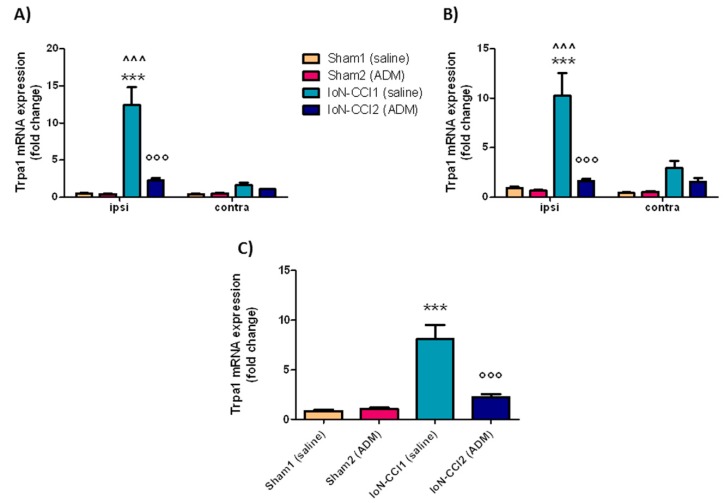
*Trpa1* mRNA expression in trigeminal ganglia (TGs) (**A**), cervical spinal cord (CSC) (**B**), and medulla (**C**). Data is expressed as mean + SEM. One way analysis of variance (ANOVA) followed by Tukey’s Multiple Comparison Test or Two-way ANOVA followed by Bonferroni post-hoc test, *** *p* < 0.001 vs. Sham1 and Sham2 (ipsi), °°° *p* < 0.001 vs. IoN-CCI1 (ipsi), ^^^ *p* < 0.001 vs. IoN-CCI1 (contra).

In the ipsilateral TG and CSC, and in medulla region, *Trpa1* mRNA expression levels were significantly increased in the IoN-CCI1 group compared with Sham1 and Sham2 groups (Figure 2). The increased mRNA levels were significantly reduced after treatment with ADM_12 in IoN-CCI rats (IoN-CCI2 group) in the same regions (Figure 2). ADM_12 administration did not provoke any changes in sham-operated rats.

A significant difference in mRNA levels, both in TG and CSC, was detected between sides in the IoN-CCI1 group; whereas, there was no difference between groups when comparing *Trpa1* mRNA levels on the contralateral side of TG and CSC (Figure 2A,B).

#### 2.2.2. *Trpv1* mRNA Expression

**Figure 3:**
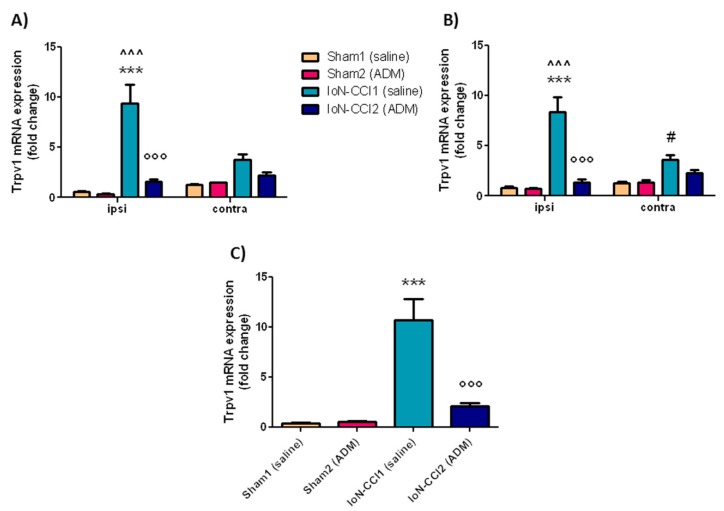
*Trpv1* mRNA expression in TGs (**A**), CSC (**B**), and medulla (**C**). Data is expressed as mean + SEM. One way analysis of variance (ANOVA) followed by Tukey’s Multiple Comparison Test or Two-way ANOVA followed by Bonferroni post-hoc test, *** *p* < 0.001 vs. Sham1 and Sham 2 (ipsi), °°° *p* < 0.001 vs. IoN-CCI1 (ipsi), ^^^ *p* < 0.001 vs. IoN-CCI1 (contra), ^#^ *p* < 0.05 vs. Sham1 and Sham2 (contra).

In the ipsilateral TG and CSC, and in the medulla region, *Trpv1* mRNA expression levels were significantly increased in the IoN-CCI1 group compared with Sham1 and Sham2 groups (Figure 3). The mRNA levels of *Trpv1* were also significantly higher in the IoN-CCI1 group in the contralateral CSC when compared to Sham groups (Figure 3B), though this increase was less marked than the increase observed on the ipsilateral side. The increased mRNA levels in these areas were significantly reduced by ADM_12 treatment in CCI rats (IoN-CCI2 group) in ipsilateral TG and CSC, and in medulla in toto (Figure 3). ADM_12 administration did not provoke any changes in sham-operated rats.

A significant difference was seen between the ipsi- and contralateral side (both in TG and CSC) in the IoN-CCI1 (Figure 3A,B).

#### 2.2.3. *Calca* mRNA Expression

**Figure 4:**
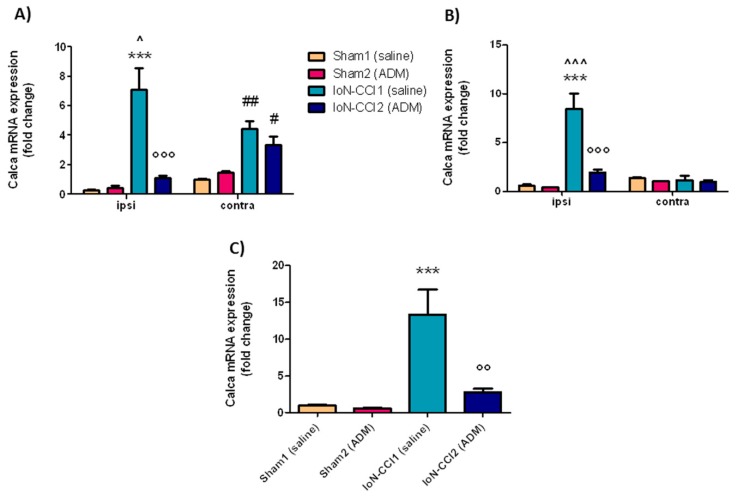
*Calca* mRNA expression in TGs (**A**), CSC (**B**), and medulla (**C**). Data is expressed as mean + SEM. One way analysis of variance (ANOVA) followed by Tukey’s Multiple Comparison Test or Two-way ANOVA followed by Bonferroni post-hoc test, *** *p* < 0.001 vs. Sham1 and Sham2 (ipsi), °° *p* < 0.01 and °°° *p* < 0.001 vs. IoN-CCI1 (ipsi), ^ *p* < 0.05 and ^^^ *p* < 0.001 vs. IoN-CCI1 (contra), ^#^ *p* < 0.05 and ^##^ *p* < 0.01 vs. Sham1 and Sham2 (contra).

In the ipsilateral TG and CSC, and in the medulla region, *Calca* mRNA expression levels were significantly increased in the IoN-CCI1 group compared with Sham1 and Sham2 groups (Figure 4). Moreover, *Calca* mRNA levels in IoN-CCI1 and IoN-CCI2 groups were also significantly increased in the contralateral TG as compared to Sham groups (Figure 4A). The increased mRNA levels were significantly reduced after treatment with ADM_12 in IoN-CCI2 rats in ipsilateral TG and CSC, and in medulla in toto (Figure 4). ADM_12 administration did not provoke any changes in sham-operated rats (Figure 4).

A significant difference was seen between the ipsi- and contralateral side (both in TG and CSC) in the IoN-CCI1 group (Figure 4A,B).

#### 2.2.4. *PPT-A* mRNA Expression

**Figure 5:**
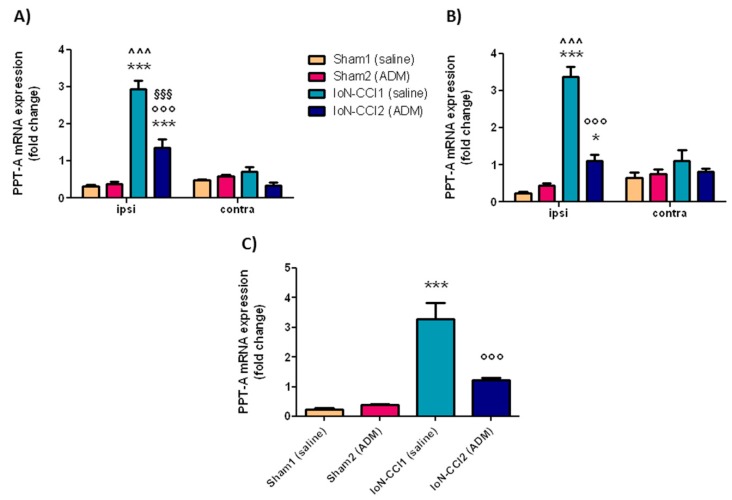
*PPT-A* mRNA expression in TGs (**A**), CSC (**B**), and medulla (**C**). Data is expressed as mean + SEM. One way analysis of variance (ANOVA) followed by Tukey’s Multiple Comparison Test or Two-way ANOVA followed by Bonferroni post-hoc test, * *p* < 0.05 and *** *p* < 0.001 vs. Sham1 and Sham2 groups (ipsi), °°° *p* < 0.001 vs. IoN-CCI1 group (ipsi), ^^^ *p* < 0.001 vs. IoN-CCI1 group (contra), ^§§§^ *p* < 0.001 vs. IoN-CCI2 (contra).

In the ipsilateral TG and CSC, and in the medulla region, *PPT-A* mRNA expression levels were significantly increased in the IoN-CCI1 group compared with Sham1 and Sham2 groups (Figure 5). The increased mRNA levels were significantly reduced after treatment with ADM_12 in IoN-CCI rats (IoN-CCI2 group) in the same regions (Figure 5). ADM_12 administration did not cause any changes in sham-operated rats.

A significant difference was seen between the ipsi- and contralateral side (both in TG and CSC) in the IoN-CCI1 group, as well as in the IoN-CCI2 group at the TG level; whereas, there was no difference between groups on the contralateral side of TG and CSC (Figure 5A,B).

#### 2.2.5. *IL-1beta*, *IL-6*, and *TNF-alpha* mRNA Expression

Since the effects of the surgery, and consequently of the TRPA1 antagonist, on the transcript levels were seen mainly at the ipsilateral side, the cytokines mRNA expression was not evaluated contralaterally. 

**Figure 6:**
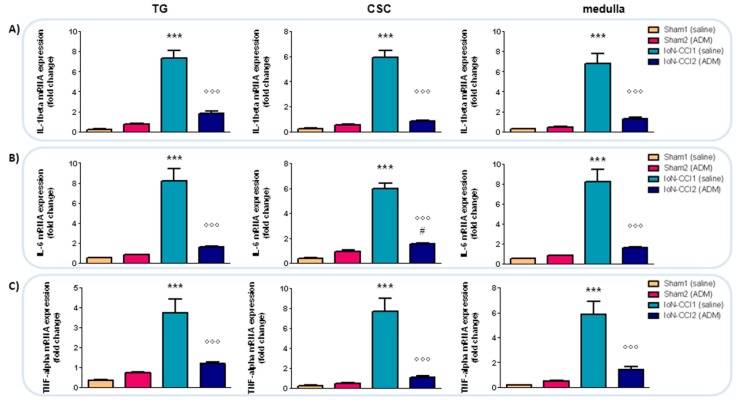
mRNA expression of *IL-1beta* (**A**), *IL-6* (**B**), and *TNF-alpha* (**C**) in ipsilateral TG and CSC, and in medulla in toto. Data are expressed as mean + SEM. One way analysis of variance (ANOVA) followed by Tukey’s Multiple Comparison Test, *** *p* < 0.001 vs. Sham1 and Sham2, °°° *p* < 0.001 vs. IoN-CCI1, ^#^ *p* < 0.05 vs. Sham1.

Interleukin *(IL)-1beta*, *IL-6*, and tumor necrosis factor *(TNF)-alpha* mRNA expression levels were significantly increased in all the areas under evaluation in the IoN-CCI1 group compared with Sham1 and Sham2 groups (Figure 6). Such increases were significantly reduced after treatment with ADM_12 in IoN-CCI rats (IoN-CCI2 group) in the same regions (Figure 6).

### 2.3. ADM_12 Effect on Neuropeptide Protein Expression

**Figure 7:**
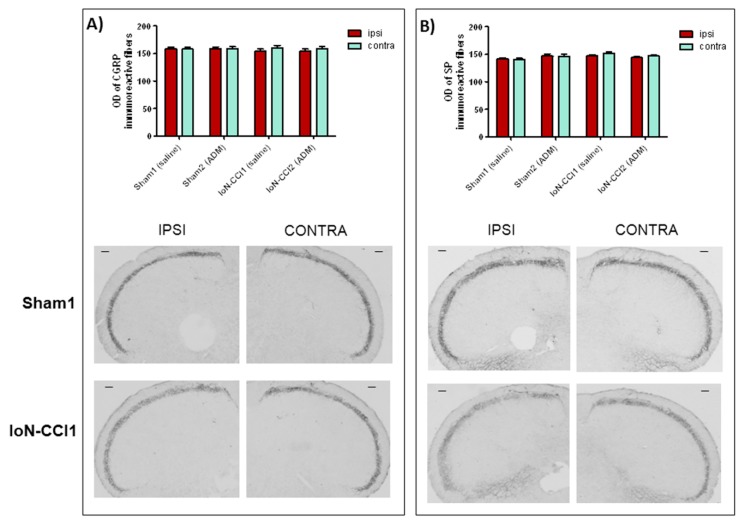
(**A**) Optical density (OD) values of calcitonin gene-related peptide (CGRP) with representative photomicrographs of CGRP immunoreactive fibers in the spinal nucleus of trigeminal nerve pars caudalis (Sp5C) ipsilateral (ipsi) and contralateral (contra) of Sham1 and IoN-CCI1 groups. (**B**) OD values of substance P (SP) with representative photomicrographs of SP immunoreactive fibers in the Sp5C ipsilateral (ipsi) and contralateral (contra) of Sham1 and IoN-CCI1 groups. Data is expressed as mean + SEM. Two-way analysis of variance (ANOVA) followed by Bonferroni post-hoc test. Scale bar: 100 µm.

CGRP and SP protein expression was evaluated in Sp5C on both sides. A slight, but not significant difference in the density of immunoreactive fibers for CGRP and SP protein was observed between the ipsilateral and contralateral side in both the IoN-CCI1 and IoN-CCI2 groups (Figure 7). No significant change was seen between sham and operated rats (Figure 7). ADM_12 administration did not induce any change in CGRP and SP expression either in sham or in CCI operated rats (Figure 7).

## 3. Discussion

The pathways of trigeminal neuropathic pain are poorly understood. Experimental evidences suggest a strong involvement of TRPA1 in different patterns of neuropathic pain, and recently its role was also demonstrated in a trigeminal neuropathic pain model [17]. 

Here we evaluated the role of TRPA1 channels in an animal model of trigeminal neuropathic pain (IoN-CCI model), investigating the effects of the TRPA1 antagonist ADM_12 on mechanical allodynia, and neurochemical and transcriptional changes. 

ADM_12 was previously shown to revert *in vivo* the Oxaliplatin-induced neuropathy [22]. At the trigeminal level, ADM_12 was able to reduce orofacial pain in a model of temporomandibular joint inflammation [23], and to counteract trigeminal hyperalgesia in a model of migraine pain, together with decreased *Trpa1* and neuropeptide mRNA expression levels in specific areas implicated in trigeminal pain [24].

### 3.1. Behavioral Response

Infraorbital nerve injury in rats leads to the development, in the ipsilateral side, of a hyporesponsiveness to mechanical stimulation within the first week post operation, followed by a hyperresponsiveness, that according to several studies [18,25], reflects a condition of mechanical allodynia. This biphasic response is probably related to the demyelination process, occurring in the early post-operative period, and remyelination process, that occurs in the late post-operative period [26]. Compared to the above cited papers [18,25,26], the time needed in this study to develop allodynia was somewhat longer. This may have been the result of small differences in the degree of nerve constriction; indeed, different degrees of IoN constriction have been shown to produce different time courses in isolated face grooming behavior [27], and this can also be true for mechanical allodynia.

The allodynic response of operated rats was abolished after treatment with the TRPA1 antagonist ADM_12, suggesting that the blockade of TRPA1 channels located on the trigeminal afferents prevented the release of neuropeptides (CGRP and SP) [28,29], thus resulting in a reduced neurogenic inflammation, and ultimately nociceptor sensitization [30]. Accordingly, Wu and colleagues reported an increase in TRPA1 protein, as well as TRPV1 channels, in the Sp5C region of rats that underwent IoN-CCI surgery [31], confirming their involvement in this process. An additional mechanism is represented by the reduction in the release of pro-inflammatory factors via the inhibition of TRPA1 located on glial cells in the nervous system, as suggested by our results, in which we observed a reduction of the *IL-1beta*, *IL-6*, and *TNF-alpha* transcripts that possibly parallel protein expression [32,33,34], which could account for reduced glial cells activation [32]; or via the inhibition of TRPA1 located on non-neuronal cells, such as keratinocytes and macrophages, in the tissues surrounding the damaged nerve [35]. Pro-inflammatory mediators released in the tissues that surround the damaged nerve, and glial cell activation, are indeed known to play a crucial role in the pathophysiology of neuropathic pain [36,37]. Glial activation and pro-inflammatory cytokines are associated with the onset of neuropathic pain symptoms such as allodynia or hyperalgesia [38,39,40,41,42]. 

The involvement of TRPA1 in mechanosensation has been extensively studied; both genetic deletion of TRPA1 and its pharmacological blockade abrogate mechanical pain-like behaviors [17,43,44]. Recently, Trevisan and colleagues [17] confirmed the critical role played by TRPA1 channels in mechanical allodynia induced by trigeminal neuropathic pain; conversely, in a model of sciatic nerve injury, Lehto and co-workers [45] reported a non-significant involvement of these channels in the mechanical sensitivity. On the other hand, other authors showed that TRPA1 blockade attenuated mechanical hypersensitivity following spinal injury [46,47] or neuropathic pain induced by chemotherapeutic agents [48,49]. Altogether these observations suggest that mechanical allodynia might be differently mediated by TRPA1 channels depending on the type of pain, site of damage, or distribution profile in TG and DRGs [50]. Moreover, the different responses observed in the experimental models could also be related to the different TRPA1 antagonists used, that may inhibit the channel through binding at different sites, with specific regulatory mechanisms [51]. 

### 3.2. Trpa1 and Trpv1 mRNA Expression

Chronic constriction injury of the IoN produced a marked increase in the *Trpa1* and *Trpv1* mRNA expression in central and peripheral areas ipsilaterally, and a slight increase even at the contralateral side, compared to the sham group. This contralateral increase is probably due to activation of inflammatory processes occurring after nerve injury, which can also affect the contralateral side [52]. The elevated TRP transcripts are accompanied by increased *IL-1beta*, *IL-6*, and *TNF-alpha* mRNA levels in the medulla region, and ipsilateral TG and CSC. It is known that TRPA1 and TRPV1 channels can be sensitized by inflammatory agents, causing up-regulation of these channels [53,54,55]. For example, *Trpa1* expression has been shown to be up-regulated by TNF-alpha and IL-1 alpha via transcriptional factor hypoxia-inducible factor-1α [56]. Similarly, TNF-alpha can up-regulate TRPV1 protein and mRNA in DRG and TG neurons [57,58]; one of the suggested pathways for *Trpv1* regulation is the p38 mitogen-activated protein kinase pathway [59], which may also be partly involved in *Trpa1* expression [60]. As regards TRPA1, its activation seems to depend on the activation of the nuclear factor-κB signaling pathway [61].

Furthermore, an important role in neuropathic pain seems to be played by oxidative stress [62,63,64], whose components can directly activate TRPA1 channels [65], thereby contributing to inflammation in a TRPA1-dependent manner. Indeed, it was recently found that trigeminal neuropathic pain behaviors were mediated by TRPA1 targeted by oxidative stress by-products released from monocytes and macrophages surrounding the site of the nerve injury [17]. 

In agreement with our study, an up-regulation of *Trpa1* and *Trpv1* mRNA levels, as well as protein levels in TG, DRGs, and dorsal horns, has been seen in different models of neuropathic pain [11,47,60,66,67,68,69,70,71]. Increased mRNA levels may reflect an increase in functional TRPA1 and TRPV1 channels [72,73].

The increased mRNA levels detected in our experiments in CSC and medulla may have different origins: *Trpv1* mRNA undergoes bidirectional axon transport along primary afferents [74], and the same could be true for *Trpa1*, since both TRPV1 and TRPA1 are (co-)expressed, not only on peripheral, but also on central terminals of primary afferent neurons where their activation can lead to the release of transmitters that promote the sensitization of postsynaptic pain transmission pathways [75,76,77,78]. In addition, *Trpv1* mRNA could originate from GABAergic interneurons and glial cells in the rat dorsal horn, which are known to express TRPV1 [69,79].

Systemic administration of ADM_12 markedly reduced the mRNA expression levels of both TRPs induced by IoN ligation. The effect of drug treatment on mRNA transcripts is likely to be due to an indirect effect rather than a direct one. It can be reasonably hypothesized that the effect of ADM_12 on TRPA1 mRNA expression is indirectly due to the blockade of the channel, located either on neuronal and non-neuronal cells, which is followed by two events. On one side, the reduction of calcium (Ca^2+^) entry provokes a reduced activation of second messenger (Ca^2+^ dependent) molecules (e.g., via the phospholipase C/Ca^2+^ signaling pathway and Ca(^2+^)/calmodulin-dependent protein kinase II [CaMKII]) and interfering with the Ca^2+^-interacting proteins [80,81], with the consequent reduction in transcriptional rate; for example, through the CaMK—cAMP response element-binding protein (CaMK—CREB) cascade. The other event that follows TRPA1 antagonism is the reduction in neuropeptide (CGRP and SP) release [28,29], and pro-inflammatory agents from neuronal fibers and non-neuronal cells. In this frame, we hypothesize that ADM_12 may break off a self-feeding loop in which TRPA1 channels are directly activated or sensitized by Ca^2+^ [51,81], endogenous substances produced by intracellular Ca^2+^ elevation [82], and pro-inflammatory molecules [83,84,85], and indirectly by the activation of nociceptive fibers caused by neuropeptide-induced neuroinflammation.

Moreover, we can also speculate that since TRPA1 and TRPV1 functions may be influenced by each other [20,86,87], a re-organization in the expression and nature of these channels after nerve injury [88,89,90] enabled ADM_12 to modulate TRPV1 channels as well. Although a physical interaction between these two channels may be questionable, even if some studies described it *in vitro* [19,21], many studies reported a functional interaction between them [20,86,91,92,93]. For instance, Masuoka et al. [87] showed in DRG neurons that TRPA1 channels suppress TRPV1 channel activity, possibly through the regulation of basal intracellular calcium concentration, and that the TRPA1 sensitization, induced by inflammatory agents, enhance TRPV1-mediated currents [87]. 

These observations, including our data, show a relationship between these two TRP channels, although more information and studies are needed to understand the precise mechanisms of this putative interaction.

### 3.3. Neuropeptide Expression

After nerve injury, an inflammatory process leads to the release of many pro-inflammatory mediators, which participate in peripheral sensitization, promoting an excessive release of neurotransmitters [94]. Together with the inflammatory process, neuropeptides and degenerative changes affecting the nervous fibers are also crucial peripheral mechanisms [95]. 

In our experimental setting, the mRNA expression levels of genes coding for CGRP (*Calca*) and SP (*PTT-A*) markedly increased in the central areas containing the Sp5C, as well as in the TG ipsilateral to the IoN ligation. Interestingly, *Calca* mRNA expression in IoN ligated rats was also elevated on the contralateral TG. It has been shown that projections from the TG reach the medullary and cervical dorsal horns on both sides [96,97], and that unilateral TG stimulation activates neurons in both ipsi- and contralateral Sp5C [98,99]. 

One of the mechanisms that could contribute to neuropeptide expression is the CaMK—CREB cascade, which is probably triggered following TRP channel activation [100], and that may represent the target mechanism for the observed inhibitory effect of ADM_12 on the mRNA expression of CGRP and SP. The blockade of TRP channels, which co-localize with CGRP and SP in the trigeminal neurons [7,101], can inhibit *Calca* and *PPT-A* mRNA expression, thus reducing the neuropeptide release and the trigeminal sensitization process. The data supports the pivotal involvement of CGRP and SP in the delivery and transmission of pain sensation to the central nervous system, and their role in trigeminal pain syndrome. In fact, an increased concentration of neuropeptides was found in the cerebrospinal fluid and venous blood of patients with trigeminal neuralgia compared to healthy controls [102,103]. 

In this frame, it was quite surprising that we did not detect any significant difference in neuropeptide protein expression at the Sp5C level, neither among groups, nor between sides. Lynds and co-workers [104] reported no differences in neuropeptide (CGRP and SP) levels between ipsi- and contralateral TG two weeks after IoN transection injury, while Xu and colleagues [105] described a reduction of CGRP and SP protein levels in the ipsilateral caudal medulla eight days after partial IoN ligation. Taken together, these findings suggest that in our model the neuropeptide release at central sites might have taken place at early time points after surgery, and therefore went undetected since we only measured it on day +27, or alternatively, that CGRP and SP are mostly involved at the peripheral terminals [26]. These apparently contrasting findings prompt the need for specifically targeted studies in order to investigate in more depth the role of neuropeptide release in central and peripheral sites in this model of trigeminal neuropathic pain.

### 3.4. Limitations of the Study and Future Perspectives

We evaluated changes of behavioral responses and mRNA expression after a short period (1 h) of drug exposure. This approach may be questionable, however there are many studies that support our observations. For instance, the mRNA expression of metabotropic glutamate receptors was found to be upregulated 1 h after treatment in mice DRG neurons [106]. Ambalavanar et al. [107] were able to detect changes in CGRP mRNA levels in rat’s TG even 30 minutes after complete Freund’s adjuvant injection. Furthermore, Nesic and co-workers [108] reported change in the mRNA signal of cytokines 1 h after treatment with MK-801, a NMDA receptor antagonist, in the spinal cord of rats subjected to spinal cord injury. 

Nevertheless, to elucidate and confirm the present findings, additional experiments with different techniques are necessary. It will be interesting to evaluate in this model the effects of ADM_12 at later time points, as well as after chronic treatment. Another limitation of the present study is the absence of a time course of the expression of CRGP and SP. This was motivated by the ethical and organizational need to keep the number of rats as low as possible. However, based on the present findings, it seems important to address in future studies the parallel evaluation of mRNA and protein expression of CGRP and SP in order to elucidate more clearly the role of these neuropeptides in peripheral and central sites.

## 4. Materials and Methods

### 4.1. Animals

Male Sprague-Dawley rats (Charles River, weighing 225–250 g at arrival) were used following the International Association for the Study of Pain (IASP)’s guidelines for pain research in animals [109]. Animals were housed in groups of 2 with water and food available ad libitum, and kept in a colony room (humidity: 45 ± 5%; room temperature: 21 ± 1 °C). Rats were kept under a reversed 12:12 h dark/light cycle (lights on at 20 h). All procedures were in accordance with the European Convention for Care and Use of Laboratory Animals, and were approved by the Ethical Committee for Animal Testing (*Ethische Commissie Dierproeven*, ECD) of the University of Antwerp (number 2017-16, approval 20/02/2017). 

Rats were allowed to acclimate for 8 days to the housing conditions before the surgery; they were habituated to the behavioral test procedure daily for three days before pre-operative testing. Habituation and testing were conducted in a darkened room (light provided by a 60 W red light bulb suspended 1 m above the observation area) with a 45 dB background noise. 

### 4.2. Surgery

The IoN-CCI was performed as previously described [18,25,27]. Rats were anaesthetized with pentobarbital (60 mg/kg, intraperitoneally (i.p.)) and treated with atropine (0.1 mg/kg, i.p.). The surgery was performed under direct visual control using a Zeiss operation microscope (×10–25). The rat’s head was fixed in a stereotaxic frame and a mid-line scalp incision was made, exposing the skull and nasal bone. The edge of the orbit was dissected free, and the orbital contents were deflected with a cotton-tipped wooden rod to give access to the left IoN, which was loosely ligated with two chromic catgut ligatures (5-0) (2 mm apart). The scalp incision was closed using polyester sutures (4-0; Ethicon, Johnson & Johnson, Belgium). In sham operated rats, the IoN was exposed using the same procedure, but the nerve was not ligated.

### 4.3. Mechanical Stimulation Testing (MST)

Baseline data were obtained 1 day before surgery. Following surgery, rats were tested on post-operative days +5, +12, +18, +26, and +27 (Figure 1). A graded series of five Von Frey hairs (0.015 g, 0.127 g, 0.217 g, 0.745 g, and 2.150 g) (Pressure Aesthesiometer®, Stoelting Co, Chicago, IL, USA) were applied by an experimenter who was blind to animal and treatment groups, within the IoN territory, near the center of the vibrissal pad [25,110,111,112,113]. Von Frey hairs were applied in an ascending order of intensity either ipsi- or contralaterally. The scoring system described by Vos [25] was used to evaluate the rats’ response to the stimulation (Table 1). For each rat, and at every designated time, a mean score for the five von Frey filaments was determined. 

### 4.4. Drug and Experimental Plan

The TRPA1 antagonist ADM_12, synthesized in the Laboratory of Prof. Cristina Nativi (University of Florence, Italy) and characterized by a high binding constant versus TRPA1 [23], was dissolved in saline and administered intraperitoneally (i.p.) at the dose of 30 mg/kg in a volume of 1 ml/kg [22,23,24]. 

The animals were randomly allocated in four groups of 12 animals each and assigned to different experimental sets, as shown in Table 2.

**Figure 8:**
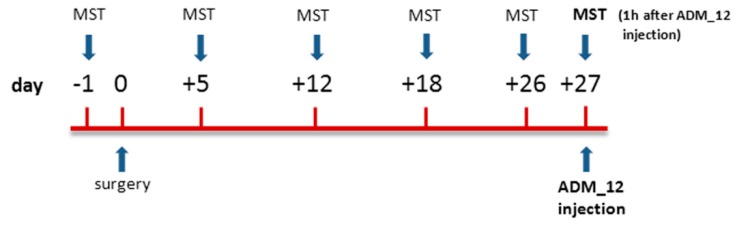
Schematic representation of the experimental design.

On day +27, sham and operated rats were treated with ADM_12 or saline 1 h prior to the MST (Figure 8). The timing was chosen on the basis of previous studies reporting a significant effect of acute ADM_12 treatment on behavioral responses [22,23,24]. At the end of the behavioral test, each rat was sacrificed with an i.p. overdose of pentobarbital (150 mg/kg). A subset of 6 rats per experimental group served for the detection of gene expression levels by means of real time polymerase chain reaction (RT-PCR); another subset of 6 animals per experimental group underwent the immunohistochemical evaluation of protein expression (Table 2).

### 4.5. Real Time-PCR

The trigeminal ganglia (TG), cervical spinal cord (CSC, C1-C2 level), and medulla (bregma, −13.30 to −14.60 mm; Paxinos and Watson 4th edition), containing the Sp5C, of each animal were quickly removed after completing the MST on day +27 and frozen at –80 °C. Samples were then processed to evaluate the expression levels of the genes encoding for TRPA1 (*Trpa1*), TRPV1 (*Trpv1*), CGRP (*Calca*), SP (*PPT-A*), IL-1beta (*IL-1beta*), IL-6 (*IL-6*), and TNF-alpha (*TNF-alpha*). mRNA levels were analyzed by RT-PCR, as previously described [24,114,115]. After tissue homogenization by means of ceramic beads (PRECELLYS, Berthin Pharma, Montigny-le-Bretonneux, France), total RNA was extracted with TRIzol® reagent (Invitrogen, Carlsbad, California, USA) and quantified by measuring the absorbance at 260/280 nm using a nanodrop spectrophotometer (Euroclone, Pero (MI), Italy). Following cDNA generation with the iScript cDNA Synthesis kit (BIO-RAD, Hercules, California, USA), gene expression was analyzed using the Fast Eva Green supermix (BIO-RAD). Primer sequences were obtained from the AutoPrime software (http://www.autoprime.de/AutoPrimeWeb) (Table 3). The amplification was performed through two-step cycling (95–60°C) for 45 cycles with a Light Cycler 480 Instrument RT-PCR Detection System (Roche, Milan, Italy). The expression of the housekeeping gene, glyceraldehyde 3-phosphate dehydrogenase (*GAPDH*), remained constant in all the experimental groups considered. All samples were assayed in triplicate.

### 4.6. Immunohistochemistry

According to Terayama et al. [116] and Panneton et al. [117], the central afferent innervations of the IoN are mostly distributed in (but not restricted to) the dorsal and lateral part of the Sp5C, projecting to all the laminae. The pattern of CGRP and SP protein related to the painful component of the IoN was investigated in the superficial laminae of the Sp5C. 

Immediately after the MST test, animals were anaesthetized and perfused transcardially with phosphate buffered saline (PBS) and 4% paraformaldehyde. The medullary segment containing the Sp5C, between +1 and −5 mm from the obex, was removed and post-fixed for 24 h in the same fixative; subsequently, samples were transferred in solutions of sucrose at increasing concentrations (up to 30%) during the following 72 h. All samples were cut transversely at 30 µm on a freezing sliding microtome. CGRP and SP protein expression was evaluated using the free-floating immunohistochemical technique, as previously reported [24]. For CGRP we used an anti-rabbit antibody (Santa Cruz Biotechnology, Santa Cruz, CA, USA) at a dilution of 1:3200, and an anti-rabbit antibody (Chemicon, Temecula, CA, USA) at a dilution of 1:5000 for SP; both primary antibodies were incubated for 24 h at room temperature. After incubation at room temperature with the secondary biotinylated antibody (Vector Laboratories, Burlingame, CA, USA) and the avidin-biotin complex (Vectastain, Vector Laboratories), sections were stained with the peroxidase substrate kit DAB (3′3′-diaminobenzidine tetrahydrochloride) (Vector Laboratories, Burlingame, CA, USA). 

The area covered by CGRP and SP immunoreactive fibers in the Sp5C ipsilateral and contralateral to the surgery (12 sections per animal), was expressed as optical density (OD) values [24,114,118], acquired using an AxioSkop 2 microscope (Zeiss) and a computerized image analysis system (AxioCam, Zeiss, Göttingen, Germany) equipped with dedicated software (AxioVision Rel 4.2, Zeiss, Göttingen, Germany). All sections were averaged and reported as the mean + SEM of OD values. 

### 4.7. Statistical Evaluation

Data from recent studies [18,27] was used to calculate the required number of animals per experimental group to obtain a statistical power of 0.80 at an alpha level of 0.05, and a difference of at least 20% in behavioral responses after IoN-CCI surgery. The calculations were done using software (Lenth RV. Java Applets for Power and Sample Size) retrieved on 8 April 2013, from http://www.stat.uiowa.edu/~rlenth/Power, which estimated a sample size of 12 rats per experimental group.

Statistical analysis was performed with the GraphPad Prism program (GraphPad Software, San Diego, California, USA). In the MST, for each rat and at every designated time, a mean score for the five Von Frey hairs was determined. The IoN-CCI rats were compared to the sham-operated rats. For mRNA levels, results were analyzed using the ΔΔ*C*t method to compare expression of genes of interest with that of *GAPDH*, used as control gene. All data was tested for normality using the Kolmogorov–Smirnov normality test and considered normal. Differences between groups, or between ipsilateral and contralateral sides, were analyzed by one-way analysis of variance (ANOVA) followed by Tukey’s Multiple Comparison Test, or by means of two-way ANOVA followed by Bonferroni post-hoc test, respectively. Differences between two groups were analyzed by the Paired student’s t test. A probability level of less than 5% was regarded as significant.

## 5. Conclusions

Antagonism of the TRPA1 channel by means of ADM_12 attenuates experimentally-induced mechanical allodynia [17,119] in a reliable animal model of trigeminal neuropathic pain. Allodynia is one of the major clinical features of trigeminal neuropathic pain [120,121], thus the modulation of the TRPA1 channel may represent a suitable therapeutic target [122,123], and ADM_12 a possible tool, in trigeminal neuropathic pain management. As a corollary, our data also suggests a possible role for TRPV1 channels in the behavioral and biomolecular responses related to trigeminal neuropathic pain. Further exploration of the mechanisms underlying the antinociceptive effects of TRPA1, and studies directed to better understand the relationship between TRPA1 and TRPV1, would improve our understanding of the complex nociceptive processing in trigeminal neuropathic pain.

## Figures and Tables

**Table 1:** Response categories with the corresponding score values.

SCORE	TYPE OF RESPONSE
**0**	no response
**1**	detection: the rat turns the head toward the stimulating object and the stimulus object is then explored
**2**	withdrawal reaction: the rat turns the head slowly away or pulls it briskly backward when the stimulation is applied; sometimes a single face wipe ipsilateral to the stimulated area occurs
**3**	escape/attack: the rat avoids further contact with the stimulus object, either passively by moving its body away from the stimulating object to assume a crouching position against the cage wall, or actively by attacking the stimulus object, making biting and grabbing movements
**4**	asymmetric face grooming: the rat displays an uninterrupted series of at least three face-wash strokes directed toward the stimulated facial area

**Table 2:** Experimental groups and number (N) of animals per group that underwent the mechanical stimulation test (MST). The samples of the subsets were processed for the real time polymerase chain reaction (RT-PCR) or immunohistochemistry (IHC).

EXPERIMENTAL GROUPS	Surgery	Treatment on Day +27	MST	RT-PCR	IHC
Sham1	Sham	saline	N = 12	N = 6	N = 6
Sham2	Sham	ADM_12	N = 12	N = 6	N = 6
IoN-CCI1	IoN-CCI	saline	N = 12	N = 6	N = 6
IoN-CCI2	IoN-CCI	ADM_12	N = 12	N = 6	N = 6

**Table 3:** Primer sequences.

Gene	Forward Primer	Reverse Primer
*GAPDH*	AACCTGCCAAGTATGATGAC	GGAGTTGCTGTTGAAGTCA
*Trpa1*	CTCCCCGAGTGCATGAAAGT	TGCATATACGCGGGGATGTC
*Trpv1*	CTTGCTCCATTTGGGGTGTG	CTGGAGGTGGCTTGCAGTTA
*Calca*	CAGTCTCAGCTCCAAGTCATC	TTCCAAGGTTGACCTCAAAG
*PPT-A*	GCTCTTTATGGGCATGGTC	GGGTTTATTTACGCCTTCTTTC
*IL-1beta*	CTTCCTTGTGCAAGTGTCTG	CAGGTCATTCTCCTCACTGTC
*IL-6*	TTCTCTCCGCAAGAGACTTC	GGTCTGTTGTGGGTGGTATC
*TNF-alpha*	CCTCACACTCAGATCATCTTCTC	CGCTTGGTGGTTTGCTAC

## Abbreviations

CaMKII	Ca(^2+^)/calmodulin-dependent protein kinase II
Calca	calcitonin-related polypeptide alpha
CGRP	calcitonin gene-related peptide
CREB	cAMP response element-binding protein
CSC	cervical spinal cord
DRG	dorsal root ganglia
GAPDH	glyceraldehyde 3-phosphate dehydrogenase
IL	interleukin
IoN	infraorbital nerve
IoN-CCI	chronic constriction injury of the infraorbital nerve
MST	mechanical stimulation testing
OD	optical density
PPT-A	preprotachykinin-A
RT-PCR	real time polymerase chain reaction
SP	substance P
Sp5C	spinal nucleus of trigeminal nerve pars caudalis
TG	trigeminal ganglia
TN	trigeminal neuralgia
TNF-alpha	tumor necrosis factor alpha
TRP	transient receptor potential
